# Evaluation of the Activities of Thyroid Hormones Among Pre- and Post-menopausal Euthyroid Women: A Cross-sectional Study from a Tertiary Care Teaching Hospital in India

**DOI:** 10.7759/cureus.4259

**Published:** 2019-03-16

**Authors:** Bhagavan Reddy Kolanu, Sabitha Vadakedath, Venugopal Boddula, Venkataramana Kandi

**Affiliations:** 1 Biochemistry, Prathima Institute of Medical Sciences, Karimnagar, IND; 2 Biochemistry, Chalmeda Anandrao Institute of Medical Sciences, Karimnagar, IND; 3 Microbiology, Prathima Institute of Medical Sciences, Karimnagar, IND

**Keywords:** thyroid hormones, thyroid stimulating hormone (tsh), tri-iodothyronine (t3), thyroid gland disorders, pre-menopausal, post-menopausal, tetra-iodothyronine (t4)

## Abstract

Introduction

Aging brings about several changes in humans that include both physiological and anatomical changes. As individuals' age, the activity of the thyroid gland and its hormones decline, causing significant metabolic disorders. Most thyroid gland disorders have been noted among young and middle-aged women. Very little is known regarding the activities of thyroid hormones among older aged women.

Methods

The study included 350 young to middle-aged pre-menopausal women between 25 and 49 years and 350 older post-menopausal women above 50 years of age. The study was conducted in the department of biochemistry, Prathima Institute of Medical Sciences (PIMS), Nagunur, Karimnagar, Telangana, India. The subjects included in the study were euthyroid (not having any signs and symptoms of thyroid disorder) and were not on any medication. The thyroid profile, including thyroid stimulating hormone (TSH), tri-iodothyronine (T_3_), and tetra-iodothyronine (T_4_), was analyzed in all the study subjects using the chemiluminescence immunoassay (CLIA) technique on a completely automated Abbott i1000SR Architect Plus instrument (Abbott Core Laboratory, Illinois‎, US).

Results

There was no statistically significant difference in thyroid hormone activities in the two age groups compared, as noted by the unpaired student's ‘t’ test. The mean serum TSH levels in the older post-menopausal women (3.39+2.45) were found to be higher than those noted in pre-menopausal women (2.60+1.31). The activities of T_3 _and T_4_ showed no difference in both groups (p=0.8397).

Conclusion

The study results clearly indicate an increase in the activities of TSH among the older-aged post-menopausal women.

## Introduction

Human life relies on a delicate balance of hormones such as estrogen, progesterone, testosterone, and many others. Women come across many physiological and anatomical changes throughout their lives, including puberty, pregnancy, and menopause, which are controlled by female sex hormones. Thyroid hormones influence the development and functioning of the reproductive system and the overall body metabolism.

Thyroid diseases mainly affect women; the incidence is five to 20 times higher in women than in men. The prevalence of thyroid diseases increases with age. In women, diseases of the thyroid gland are among the most prevalent disorders worldwide, second only to diabetes [1].

Thyroid diseases are more common in middle-aged and older post-menopausal women. The diagnosis and interpretation of thyroid function tests, including an estimation of the activities of thyroid stimulating hormone (TSH), tri-iodothyronine (T_3_), and tetra-iodothyronine (T_4_) in old-age people are very difficult [2].

Most professional organizations recommend the screening of older women for thyroid dysfunction. The American Thyroid Association (ATA), the Endocrine Society and the American Association of Clinical Endocrinologists (AACE) had recommended aggressive case finding in elderly women [3]. According to the 2012 clinical practice guidelines formulated by the AACE and ATA, serum TSH is the best screening test for the primary diagnosis of thyroid disorders [3]. TSH is the preferred test to assess thyroid function, as stated by the National Academy of Clinical Biochemistry (NACB) [4].

Thyroid functions can be influenced by nutritional status, associated co-morbidities, co-factors such as body surface area, and others [5-6]. A regular follow-up of the activities of TSH could help in the clinical diagnosis and better management of the patients. According to the available literature, serum TSH concentrations may probably be age-dependent. Increased levels of serum TSH in the elderly, mainly in women, can be physiological or pathological [7-8].

This comparative cross-sectional study was undertaken to measure the serum TSH, T_3_, and T_4_ activities in young, middle-aged pre-menopausal women and post-menopausal older-aged women.

## Materials and methods

This prospective study included 350 young and middle-aged pre-menopausal women (20-49 years) and 350 elderly post-menopausal women (>50 years). All the subjects enrolled in the study were female patients attending the outpatient department of medicine at Prathima Institute of Medical Sciences (PIMS), Karimnagar, Telangana, India. Informed and oral consent was obtained from all the study participants and the study was approved by the institutional ethical committee.

All the subjects included in the study had no clinical symptoms of thyroid disorder and were not on any medication. Patients with a history of major chronic illnesses, e.g. diabetes mellitus, hypertension, other endocrinal disorders, patients on hormone replacement therapy or on drug altering serum TSH, hysterectomy patients, pre-pubertal age women, pregnant woman, and premature menopausal women were excluded from the study.

Five milliliters of venous blood was drawn aseptically from all the study subjects and serum TSH, T_3_, and T_4 _were measured by using chemiluminescence/magnetic particle immunoassay (CLIA) using an automated analyzer (Abbott i1000SR Architect Plus). The serum concentrations of TSH, T_3_, and T_4_ were expressed in μIU/ml and interpreted according to the manufacturer's recommendations.

## Results

Post-menopausal women showed slightly increased TSH activity (3.39±2.45) as compared to pre-menopausal women (2.60±1.31). The activities of T_3_ and T_4_, however, revealed no variation among the study groups. Table 1 shows the serum activities of TSH, T_3_, and T_4_ among both study groups.

**Table 1:** Thyroid hormone activities among the pre -and post-menopausal women TSH: Thyroid stimulating hormone; T_3_: Tri-iodothyronine; T_4_: Tetra-iodothyronine

Thyroid hormones	Pre-menopausal women	Post-menopausal women	Normal range	p-value
	(Mean±SD)	(Mean±SD)		
TSH	2.60±1.31	3.39±2.45	0.35-4.94µIU/mL	0.097
T_3_	1.03±0.24	0.99±0.19	0.64-1.52 ng/mL	0.551
T_4_	7.81±1.29	7.88±1.26	4.87-11.72 µg/dL	0.839

## Discussion

In the present study, we encountered mean serum TSH activities in older post-menopausal women (3.39±2.45 µIU/mL) that were comparatively higher than those observed among young, middle-aged pre-menopausal women (2.60±1.35 µIU/mL). However, the difference was not statistically significant. Multiple causes were proposed for increased TSH activities in the elderly, including nutritional iodine supply, sleep disturbances, altered sleep patterns, and others [9]. Aging is associated with changes in the pituitary-thyroid axis and there was a progressive shift in the serum TSH activities with increasing age [10]. The endocrine system undergoes changes with aging even in the absence of overt disease. Thus, an age-related fall in T_4_ activities and the reduced responsiveness of the thyroid to TSH could result in increased TSH secretion [11].

Another possibility of increased TSH activities may be due to occult thyroid disease in older people [12]. With aging, the variations in the activities of serum TSH may not be too significant as compared to the activities of other endocrine hormones, such as the ones secreted by the adrenal gland, as noted in previous studies [13].

Rojas LV et al. found higher TSH activities in post-menopausal women (2.80 µIU/mL) as compared to pre-menopausal (2.52 µIU/mL). They found an average increase in TSH values with age, although the change between groups was not significant, which was similar to the results observed from the current study. It has been suggested that an evaluation of baseline TSH levels within a group and in a defined geographical location may be significant [14].

A large, population-based study conducted by the National Health And Nutrition Examination Survey (NHANES) found higher TSH activities in women in the older age group [15]. An analysis of NHANES III (2007) showed that age-related shifts in TSH activities were not significantly altered when individuals with antithyroid antibodies were excluded from the study. Alterations in the activities of thyroid hormones under the influence of food (soy) were also previously reported [16]. Aging could influence the activities of various hormones that include the growth hormone, growth hormone-releasing hormone (GHRH), estrogen, progesterone, androgens, follicle-stimulating hormone, insulin-like growth factor 1, and others [17]. Therefore, it is important to understand the activities of thyroid hormones with aging.

Other previous studies showed completely contrasting results, where the serum TSH activities among the post-menopausal and older women were lower as compared to the pre-menopausal women who had increased activities of serum TSH [18-20]. These studies have suggested that this type of scenario may be age-related and could have been influenced by the pituitary gland.

Not many previous studies have evaluated the activities of thyroid hormones among euthyroid women. A recent study from the USA had evaluated the relationship between the thyroid hormonal activities and the menstrual function outcomes among the euthyroid women. This study had noted that the thyroid hormonal activities were influencing the menstrual function outcomes and the sex steroid hormonal activities, suggesting its potential relation to fertility [21].

Another recent research from India reported a study that included 301 euthyroid, pre-, and post-menopausal women suffering from type 2 diabetes. This study selected non-obese, obese, and overweight type 2 diabetes patients and evaluated their thyroid hormonal activities. The study observed a statistically significant (p<0.01) relation of the activities of T_4_ and TSH within the pre and post-menopausal women [22].

The evaluation of the activities of thyroid hormones among the pre-menopausal women was suggested in a previous study. This study had correlated the possibility of early atherosclerosis and the activities of TSH among the euthyroid women with autoimmune thyroiditis. This study had suggested that although euthyroid, there is a possibility of developing cardiovascular disease with the increased activities of TSH [23].

Probable mechanisms and contributing factors of thyroid dysfunction 

As evidenced by the current literature, there is a possibility of variation in the activities of thyroid hormones among women. Experimental studies in the past have highlighted the potential role of estrogen in the development of thyroid dysfunction. Studies found that estrogen receptors, along with their isoforms, on thyroid cells could modulate thyroid function, especially causing cancer [24-25].

The most accepted and probably the potential mechanism by which estrogen causes thyroid dysfunction among women, especially post-menopausal women, is its binding to the thyroglobulin. This restricts the entry of thyroxine into the cells, thereby increasing the concentrations of bound thyroxine and reducing the availability of free thyroid hormones [26].

Thyroid disease among the post-menopausal women was also attributed to the environmental toxicity caused by an increase in polybrominated diphenyl ethers (PBDEs), as evidenced by a study by Allen JG et al. from the United States of America (USA) [27].

Thyroid disease in the elderly was also attributed to autoimmunity, drugs, radiotherapy, and surgery. The activities of TSH among the elderly were found to be influenced by drugs that include glucocorticoids, somatostatin analogs, dopamine agonists, metformin, antiepileptics, lithium carbonate, and iodine-containing drugs. Other drugs, such as antacids, amiodarone, estrogens, mitotane, fluorouracil, phenobarbital, and rifampin, were also found to cause thyroid dysfunction [28]. Chronic exposure to bisphenol A (BPA), an organic compound used in making plastic, was also found to cause endocrinal abnormalities affecting human health [29]. Although its role in causing thyroid dysfunction is not yet confirmed, future studies on the potential role of BPA in causing thyroid diseases appear to be important, in view of the extensive use of plastics in developing countries like India.

Thyroid disease was also attributed to the reactivation of Epstein-Barr virus (EBV) in a recent study from Japan. This study had noted that during EBV reactivation, thyrotropin receptor antibodies (TRAbs) are formed, which in turn stimulates the TSH receptors and causes thyroid dysfunction [30].

## Conclusions

In view of the results obtained from the current research and considering the previous reports, there is a possibility of a shift in the activities of thyroid hormones with age. Considering the improved life expectancy and the fact that the activities of thyroid hormones could influence the reproductive and other metabolic pathways, it is important for us to have a better understanding of the activities of thyroid hormones as a person ages for appropriate management. Also, there is a need to understand the baseline thyroid function by measuring the activities of thyroid hormones in euthyroid women at various climacteric stages of life, including pre-puberty, after puberty, pre-pregnancy, after pregnancy, and post-menopause to determine subclinical thyroid disease/thyroid dysfunction.
